# Endoplasmic Reticulum Calcium Pumps and Tumor Cell Differentiation

**DOI:** 10.3390/ijms21093351

**Published:** 2020-05-09

**Authors:** Bela Papp, Sophie Launay, Pascal Gélébart, Atousa Arbabian, Agnes Enyedi, Jean-Philippe Brouland, Edgardo D. Carosella, Homa Adle-Biassette

**Affiliations:** 1 Institut National de la Santé et de la Recherche Médicale, UMR U976, Institut Saint-Louis, 75010 Paris, France; 2 Institut de Recherche Saint-Louis, Hôpital Saint-Louis, Université de Paris, 75010 Paris, France; 3 CEA, DRF-Institut Francois Jacob, Department of Hemato-Immunology Research, Hôpital Saint-Louis, 75010 Paris, France; edgardo.carosella@cea.fr; 4 EA481, UFR Santé, Université de Bourgogne Franche-Comté, 25000 Besançon, France; sophie.launay@univ-fcomte.fr; 5 Department of Clinical Science-Hematology Section, Haukeland University Hospital, University of Bergen, 5021 Bergen, Norway; pascal.gelebart@uib.no; 6 Laboratoire d’Innovation Vaccins, Institut Pasteur de Paris, 75015 Paris, France; atousa.arbabian@pasteur.fr; 7 Second Department of Pathology, Semmelweis University, 1091 Budapest, Hungary; enyedi.agnes@med.semmelweis-univ.hu; 8 Institut Universitaire de Pathologie, Centre Hospitalier Universitaire Vaudois, 1011 Lausanne, Switzerland; jean-philippe.brouland@chuv.ch; 9 AP-HP, Service d’Anatomie et Cytologie Pathologiques, Hôpital Lariboisière, 75010 Paris, France; homa.adle@aphp.fr; 10 Université de Paris, NeuroDiderot, Inserm UMR 1141, 75019 Paris, France

**Keywords:** SERCA, endoplasmic reticulum, calcium signaling, differentiation, cancer, leukemia, ion transport

## Abstract

Endoplasmic reticulum (ER) calcium homeostasis plays an essential role in cellular calcium signaling, intra-ER protein chaperoning and maturation, as well as in the interaction of the ER with other organelles. Calcium is accumulated in the ER by sarco/endoplasmic reticulum calcium ATPases (SERCA enzymes) that generate by active, ATP-dependent transport, a several thousand-fold calcium ion concentration gradient between the cytosol (low nanomolar) and the ER lumen (high micromolar). SERCA enzymes are coded by three genes that by alternative splicing give rise to several isoforms, which can display isoform-specific calcium transport characteristics. SERCA expression levels and isoenzyme composition vary according to cell type, and this constitutes a mechanism whereby ER calcium homeostasis is adapted to the signaling and metabolic needs of the cell, depending on its phenotype, its state of activation and differentiation. As reviewed here, in several normal epithelial cell types including bronchial, mammary, gastric, colonic and choroid plexus epithelium, as well as in mature cells of hematopoietic origin such as pumps are simultaneously expressed, whereas in corresponding tumors and leukemias SERCA3 expression is selectively down-regulated. SERCA3 expression is restored during the pharmacologically induced differentiation of various cancer and leukemia cell types. SERCA3 is a useful marker for the study of cell differentiation, and the loss of SERCA3 expression constitutes a previously unrecognized example of the remodeling of calcium homeostasis in tumors.

## 1. Introduction

### 1.1. ER Calcium Homeostasis and SERCA

Calcium signaling is based on changes of cytosolic and intra-organellar free calcium ion concentrations. Calcium ions can bind non-covalently to, and thereby allosterically modify, specific polypeptide sequences in target proteins. The ensuing conformational changes can modify the enzymatic activity and other functional characteristics of these proteins. The reversible, calcium concentration-dependent modulation of the three-dimensional structure of specific intracellular enzymes and other proteins constitutes the basis of intracellular calcium-dependent signal transduction. This requires that the calcium concentration in various cellular compartments be maintained at precisely set levels in resting cells, while permitting rapid reversible changes during cell activation [1].

**Figure 1:**
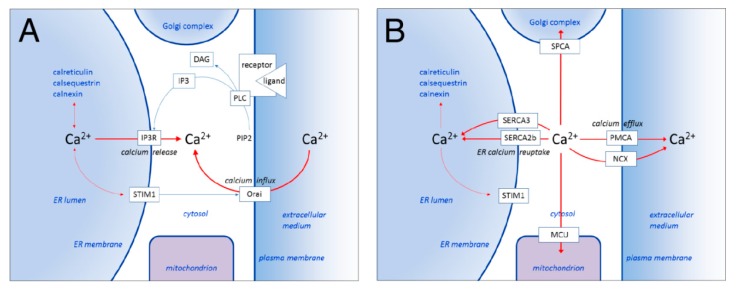
A simplified scheme of cellular calcium compartments, transporters and calcium ion fluxes. (**A**) Calcium mobilization during a cell activation event. Upon activation of plasma membrane receptors, phospholipase C (PLC)-induced hydrolysis of the membrane lipid phosphatidylinositol 4,5-bisphosphate (PIP2) leads to the formation of the second messengers diacylglycerol (DAG) and inositol 1,4,5-trisphosphate (IP3). Binding of IP3 on IP3-receptor calcium channels located in the ER membrane leads to channel opening and passive diffusion of calcium ions from the ER lumen into the cytosol (calcium release). Dissociation of calcium ions from the intraluminal part of STIM proteins located in the ER membrane leads to their interaction with Orai-type calcium channels located in the plasma membrane and Orai channel opening. Calcium influx through Orai channels (capacitative calcium influx) combined with calcium release from the ER leads to increased cytosolic calcium levels and the activation of calcium-dependent downstream signaling. (**B**) Elimination of cytosolic calcium ions after a cell activation event. Cytosolic calcium levels are decreased by the concerted action of SERCA, SPCA and PMCA-type calcium pumps, sodium/calcium exchangers (NCX) and the mitochondrial calcium uniporter complex (MCU). In non-muscle cells calcium is actively taken up in the ER by SERCA2b and SERCA3-type calcium pumps, and calcium (as well as manganese) transport into the Golgi complex is performed by SPCA-type pumps. Cytosolic calcium can also be taken up by mitochondria through the MCU and is eliminated from the cell through PMCA-type calcium pumps and sodium/calcium exchangers (NCX) into the extracellular medium that contains calcium ions in the low millimolar range. The concerted action of these mechanisms leads to the establishment of resting cytosolic calcium levels (low nanomolar). Calcium sequestered in the ER (in the high micromolar concentration range) can interact with STIM proteins, leading to inactivation of capacitative calcium influx, as well as with calcium-binding ER chaperones such as calreticulin.

The endoplasmic reticulum (ER) plays a central role in calcium signaling as a source of calcium that is released into the cytosol upon cell activation [2]. Whereas the ER lumen contains calcium ions in the high micromolar range (~100 µM), in a resting cell the cytosolic calcium concentration is low nanomolar (50–100 nM). During activation, calcium is released from the ER into the cytosol via calcium channels such as the inositol 1,4,5-trisphosphate receptor (IP3 receptor), leading to increased cytosolic calcium levels and partial ER calcium depletion [3]. ER calcium depletion then induces, via STIM proteins, calcium influx (called store operated calcium entry) into the cytosol from the extracellular space, across the plasma membrane through Orai-type calcium channels [4,5]. The calcium release from the ER combined with store operated calcium influx leads to increased cytosolic calcium levels and consequent activation of cytosolic calcium-dependent enzymes [6] (Figure 1A). In addition, calcium plays an important role also in signal transmission between the ER and other organelles [7]. In summary, the state of calcium-dependent activation of a cell is critically determined by the state of ER calcium filling and the direction and intensity of the net calcium ion flux that occurs across the ER membrane at a given time [2].

While calcium release from the ER is accomplished by various calcium channel proteins such as the IP3 receptor [2,8], calcium is accumulated in the ER by sarco/endoplasmic reticulum calcium ATPases (SERCA-type calcium pumps). Located in the ER membrane, these enzymes translocate calcium ions from the cytosol into the ER lumen by active transport, dependent on energy obtained by ATP hydrolysis [9,10,11]. The conformational changes taking place during the catalytic cycle of the enzyme have been elucidated by the detailed structural analysis of the protein [9,12,13,14,15,16,17,18]. Calcium transport by SERCA enzymes constitutes the sole mechanism whereby this ion is accumulated in the ER, and the more than a thousand-fold calcium concentration gradient between the ER lumen and the cytosol is thereby generated. By making calcium available in the ER, SERCA enzymes are therefore indispensable for calcium release from the ER for initiating calcium-dependent cell activation.

The cytosolic calcium concentration in a living cell is controlled by SERCA enzymes in concert with PMCA-(plasma membrane calcium ATPase) and SPCA (secretory pathway calcium ATPase)-type calcium pumps, sodium-calcium exchangers (NCX) and the mitochondrial calcium uniport complex (MCU). Whereas PMCA enzymes and NCX eliminate calcium ions from the cytosol into the extracellular space across the plasma membrane [19,20], calcium is taken up from the cytosol into the Golgi complex by SPCA [21,22] and into the mitochondria by the MCU [23,24] (Figure 1B). Because the regulation by cytosolic calcium levels of the transport activity of these proteins can differ, their simultaneous action during a calcium-dependent cell activation event can generate highly dynamic situations in terms of calcium transport, where the relative contribution of individual transport proteins may change depending on the evolution of the cytosolic calcium concentration. This can lead to crosstalk, partial redundancy and compensatory phenomena among calcium transporters. Moreover, the existence of complex positive and negative feedback mechanisms that operate, via calcium, between various calcium mobilization and elimination systems can lead to the generation of highly dynamic oscillatory calcium signals that can convey information selectively to different downstream effectors in the cell [25,26,27,28,29,30,31]. The precise understanding of the contribution of individual components of the cellular calcium homeostatic network in a living cell will require sophisticated in silico modeling [32,33,34,35,36,37,38,39,40,41,42,43,44] combined with further calcium concentration measurements in various intracellular compartments in cells in a quiescent state and also during various stages of calcium dependent activation, in which individual calcium transporters have been selectively inactivated or invalidated, while the expression levels of the other components, such as calcium pumps, exchangers and channels are not changed.

Moreover, calcium in the ER lumen is required also for calcium-dependent intra-ER chaperone functions involved in the maturation of newly synthesized proteins that travel across this organelle [45,46,47]. Thus, excessive ER calcium depletion can compromise protein maturation in the ER, leading to various compensatory stress responses and possible cell death [48,49,50]. In addition, because SERCA enzymes are active also during the calcium release events (limiting net calcium release), SERCA-dependent calcium transport can blunt the amplitude and the duration of cytosolic calcium transients and can modify the characteristics (amplitude, frequency, waveform) of oscillatory cytosolic calcium signals [33,51,52,53,54,55,56,57], which in turn will influence calcium-dependent cell activation [58,59,60,61]. The modulation of calcium signals by SERCA-dependent calcium sequestration constitutes a unique mechanism for the control of calcium-dependent cell activation.

### 1.2. SERCA Pharmacology

SERCA function has been explored pharmacologically using inhibitors such as thapsigargin [62], cyclopiazonic acid [63] or 2,5-di-*tert*-butyl-1,4-benzohydroquinone [64]. SERCA inhibition leads to calcium leakage from the ER into the cytosol. Using these molecules it has become clear that SERCA inhibition can lead to ER stress [65,66], apoptosis [67,68,69,70], autophagy [71], the inhibition of cell growth [72], enhancement of cell proliferation [73] or differentiation [74], protection from apoptosis [75], inhibition of the synthesis or the activity of mature oncoproteins [76,77,78,79,80,81], as well as tumor promotion [82], Epstein-Barr virus lytic reactivation [83] or induction of retrovirus expression in latently infected cells [84], the effects depending on cell type and the extent of SERCA inhibition. Thapsigargin-derived inactive prodrugs that allow the proteolytic release of active thapsigargin analogues in the proximity of tumor cells are being explored for the targeted therapy of prostate carcinoma and other malignancies [85,86,87,88]. It has also been reported that several pollutants, drugs and drug-like molecules can, in addition to their main pharmacological activities, modulate (inhibit or activate) SERCA enzymes [89,90,91,92,93,94,95,96,97,98,99,100,101,102,103,104,105,106,107,108,109,110,111,112,113,114,115,116,117,118,119,120,121,122]. This may be helpful to better understand the toxicity, the pharmacology and the side effects of these molecules and raises the possibility that some of these molecules may be repurposed for their SERCA inhibitory activity, or may serve as leads for the development of new, clinically useful selective SERCA inhibitors. For such drugs to be successfully developed it will be necessary to take into account the SERCA composition of the targeted cell types.

### 1.3. SERCA Isoenzymes

Despite the involvement of SERCA-dependent calcium transport in a multitude of physiological and pathological situations, it has been largely assumed that SERCA pumps in non-muscle cells accomplish their functions in a constitutive manner, re-establishing resting cellular calcium levels in a housekeeping enzyme-like fashion. However, it has become clear that SERCA expression can display cell type-dependent characteristics [123], that various SERCA species may co-exist in a cell [124,125,126] and that complex regulatory mechanisms of pathophysiological relevance exist based on the modulation of the activity [51,70,81,118,125,126,127,128,129,130,131,132,133,134,135,136,137,138,139,140,141,142,143,144,145,146,147,148,149,150,151] and the expression [74,117,126,132,139,140,142,143,146,147,148,150,151,152,153,154] of various SERCA-type calcium pumps.

Three SERCA genes (ATP2A1, 2 and 3) are known in humans that by alternative splicing can give rise to several isoforms. The expression of the various SERCA isoenzymes is tissue-dependent and developmentally regulated [155]. Whereas the SERCA1a and SERCA1b isoforms are expressed in adult and neonatal fast twitch skeletal muscle [156], respectively, SERCA2a is the major isoform expressed in slow twitch skeletal and cardiac muscle [156], and SERCA2b is expressed ubiquitously in all non-muscle cell types investigated so far [157]. For SERCA3 six alternative splice isoforms have been identified that differ in the C-terminal region of the proteins [126,141,144,147,149,158,159,160,161,162].

### 1.4. Co-Expression of Various SERCA Isoenzymes

**Figure 2:**
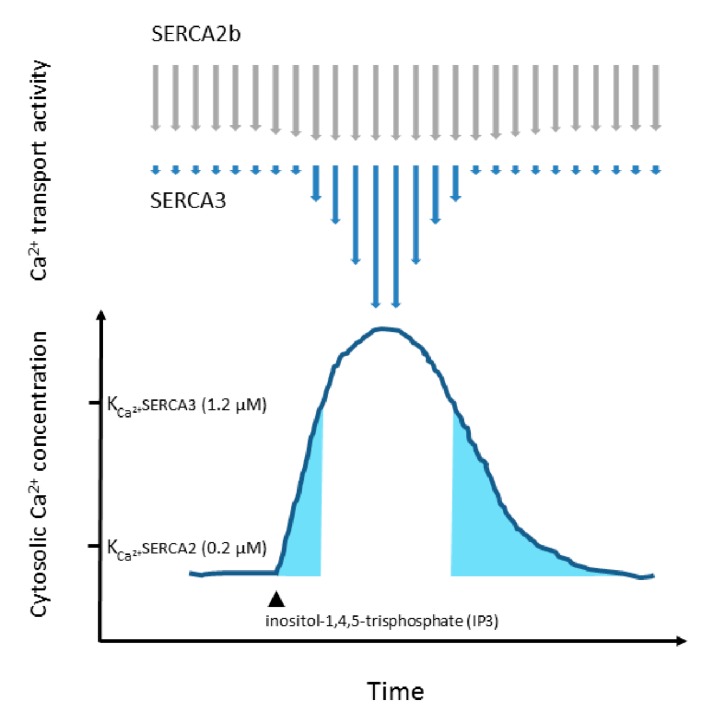
Schematic representation of the evolution of SERCA2b and SERCA3 transport activities during an IP3-induced calcium release event from the endoplasmic reticulum. Calcium transport activity (the number of calcium ions transported by a pump molecule per unit time) of the pumps is stimulated by increasing calcium concentrations. However, due to its higher calcium affinity (K_Ca_^2+^ ~ 0.2 µM), this stimulation occurs at lower calcium concentrations for SERCA2b than for the lower calcium affinity SERCA3 pump (K_Ca_^2+^ ~ 1.2 µM). The calcium affinity of SERCA2b lies closer to the calcium concentration of a resting cell, whereas that of SERCA3 is closer to calcium levels that occur during activation, in particular in specialized ER microdomains, where calcium release is locally taking place. Consequently, whereas SERCA2b is nearly maximally active already in a resting cell, SERCA3-dependent transport is at that point only weakly active, becoming activated only later, during the peak of a calcium release event (when local cytosolic calcium concentrations near the ER approaches 1.2 µM). Note that at the beginning and at the end of the calcium signal (blue areas) neither SERCA2b nor SERCA3-dependent transport is stimulated significantly by calcium. When SERCA2b is replaced by SERCA3 during cell differentiation, calcium release is facilitated, allowing more robust calcium signals. The scenario is depicted in the absence of capacitative calcium influx (in the absence of extracellular calcium) for simplicity. Upward facing arrow: cytosolic calcium concentration, downward facing arrows: SERCA calcium transport intensity. Arrow lengths depict transport intensity relative to maximally stimulated (longest arrows); SERCA2b: grey, SERCA3: blue.

In a selected set of specialized cell types (breast, colon, gastric surface epithelium, vascular endothelial cells, Purkinje neurons, β cells of islets of Langerhans, choroid plexus, and various cells of hematopoietic origin), SERCA2b and SERCA3-type calcium pumps are expressed simultaneously [11,124,125,132,161,163,164,165]. The biological rationale for the co-expression of SERCA2 and SERCA3-type calcium pumps in such a phenotypically and functionally rather disparate set of cell types is as yet unknown. SERCA2b and SERCA3 display, however, non-redundant functional characteristics. A major difference between SERCA2b and SERCA3 enzymes is that the calcium affinity of SERCA3 enzymes is significantly inferior to that of SERCA2b [161,166,167,168,169]. Due to the cooperative binding of two calcium ions to the pumps, calcium transport by SERCA enzymes is strongly activated by increasing calcium concentrations at the cytosolic side of the enzymes [170,171,172]. However, the calcium dependency of this activation is different for SERCA2b vs. SERCA3-type enzymes. Whereas half maximal calcium transport intensity for SERCA2b is attained at around a 0.2 µM free calcium ion concentration [169], half maximal activation of SERCA3 by calcium is reached only at approximately 1.2 µM calcium [158,161,167]. This means that in a resting cell SERCA-dependent calcium transport will be accomplished mainly by SERCA2b, whereas during cell activation, when cytosolic calcium levels increase, SERCA3-dependent calcium transport will be stimulated [167,168] (Figure 2). This means that SERCA3 will tolerate larger calcium increases during an ER calcium mobilization event, and this may contribute to enhanced downstream signaling and stronger effector responses. Thus, SERCA3 can be considered a less “stringent” calcium pump than SERCA2b, which may allow larger calcium signals during a cell activation event [167,168].

Humans carrying mutations in one SERCA2 allele develop Darier disease, a dermatological condition characterized by impaired keratinocyte adherence and maturation [155,173], whereas mouse heterozygous SERCA2 knock-out leads to increased incidence of tumors, including carcinomas, of the keratinizing epithelium [174,175]. SERCA3 homozygous knock-out has been shown to lead to subtle phenotypes related to vascular endothelial or bronchial epithelial cell-regulated smooth muscle cell relaxation and subtle changes in β-cell function [53,175,176,177,178,179] or platelet activation [180]. These observations show that SERCA2 and SERCA3-type calcium pumps accomplish non-redundant functions when co-expressed in a cell.

## 2. Loss of SERCA3 Expression in Tumors

Various fully mature epithelial cell types such as gastric surface epithelium, colonic and bronchial epithelial cells, breast acinar and choroid plexus epithelial cells express SERCA3 protein abundantly, whereas in corresponding tumoral tissue SERCA3 expression is strongly decreased or lost [126,139,140,142,146,150]. The widespread character of SERCA3 loss in different types of tumors indicates that a general underlying mechanism may exist whereby calcium homeostasis is remodeled during carcinogenesis. In order to explore this, SERCA3 expression was investigated in neoplasia in function of tumor type, histological grade and degree of malignant potential in situ and in the context of the underlying molecular oncogenic mechanisms in vitro. In particular, in order to better characterize features that distinguish ER calcium homeostasis of normal versus neoplastic cells, SERCA3 expression and ER calcium homeostasis have been investigated in various models of tumor/leukemia cell differentiation whereby, following various types of molecularly targeted treatments, fully malignant cells transition towards a differentiated, non-proliferating phenotype. Since the last time this was summarized [181], progress has been made in this field in several other tumor types, such as lung and breast carcinoma, benign and malignant choroid plexus tumors or acute B-cell lymphoblastic leukemia. As detailed below, by conducting such experiments on multiple types of neoplasia, it has become possible to identify a general trend that connects ER calcium transport to tumor biology, due to the down-regulation of SERCA3 expression in several types of malignancies.

### 2.1. Acute Promyelocytic Leukemia

Acute promyelocytic leukemia (APL) cells and cell lines such as NB4 and HL-60 can be induced to undergo terminal neutrophil granulocytic differentiation by all-*trans* retinoic acid (ATRA). ATRA-induced differentiation constitutes the first example of clinically efficient targeted anti-leukemia therapy [182,183]. ATRA treatment targets the PML-RARα fusion oncoprotein that blocks the differentiation of myeloid precursors at the promyelocytic stage of neutrophil granulocytic differentiation and drives APL [184,185,186]. Following ATRA treatment the cells stop proliferating and acquire several morphological as well as immunophenotypic and functional characteristics of mature neutrophil granulocytes such as lobulated nuclei, CD11b expression and the acquisition of phagocytic and NADPH oxidase activity [148,187]. During ATRA-induced differentiation, the expression of SERCA3 is induced approximately three-fold [148]. As studied in the HL-60 cell line, the induction of SERCA3 expression by ATRA was accompanied by enhanced SERCA3-dependent calcium accumulation in membrane vesicles prepared from ATRA-differentiated cells when compared to untreated control, whereas SERCA2b protein levels and SERCA2b-dependent calcium accumulation were decreased. Thus, although total calcium transport activity was not modified significantly, ATRA treatment led to a shift towards SERCA3-dependent calcium transport. This was determined using the PL/IM430 SERCA3-specific monoclonal antibody, which selectively inhibits SERCA3-dependent calcium transport. When calcium transport was measured in microsomal membrane preparations prepared from untreated and ATRA-differentiated HL-60 cells, it was found that whereas in untreated cells SERCA3-dependent transport accounted for approximately 30% of total SERCA-dependent calcium uptake, this value increased to approximately 60% following ATRA-induced differentiation [148].

In order to investigate whether changes in SERCA-dependent calcium transport are a simple passive consequence of ATRA-induced differentiation or whether SERCA activity can influence this differentiation process, HL-60 and NB4 cells were treated with increasing concentrations of SERCA inhibitors such as thapsigargin, cyclopiazonic acid or 2,5-di-*tert*-butyl- 1,4-benzohydroquinone in the presence of low concentrations of ATRA, and cell differentiation was compared to that obtained by a maximally efficient concentration of ATRA. In parallel, calcium release from the ER as well as capacitative calcium influx induced by various concentrations of SERCA inhibitors were measured using calcium spectrofluorimetry. Calcium release and capacitative calcium influx values were expressed as percentage of values obtained by maximally effective concentrations of the respective SERCA inhibitors. It was found, that inhibition of SERCA-activity that induces an approximately 60% calcium release from the ER, as well capacitative calcium entry from the extracellular space, strongly enhanced the differentiation process induced by low concentrations of ATRA [74], and resistance to ATRA-induced differentiation could also be reversed in ATRA-resistant cells by SERCA inhibitors [74]. ATRA-induced cell differentiation could be enhanced also by ritodrine [74], a clinically relevant β_2_-adrenergic agonist that possesses SERCA inhibitory activity [94].

As mentioned earlier, the calcium affinity of SERCA3 is weaker than that of SERCA2b. The replacement of SERCA2b by SERCA3 during ATRA-induced differentiation is therefore compatible with the notion of a net decrease of the calcium affinity of the ER calcium sequestration machinery during differentiation, rendering the ER more tolerant towards calcium release. Considering that the partial pharmacological inhibition of SERCA-dependent calcium transport has an analogous effect on ER calcium sequestration because this also facilitates calcium leak from the ER, and because cytosolic calcium is known to enhance ATRA-induced differentiation of HL-60 cells [188], it is tempting to hypothesize that the ATRA-induced isoenzyme switch, resulting in more SERCA3 over SERCA2b, will help the differentiation process by rendering calcium more easily available in the cytosol and by increasing cytosolic calcium levels, ultimately enabling various calcium-dependent differentiation-enhancing signaling processes.

These observations when taken together indicate that the induction of SERCA3 expression is part of the differentiation program of acute promyelocytic leukemia cells, that a functional crosstalk exists between ER-dependent calcium sequestration and the control of acute promyelocytic leukemia cell differentiation, and illustrate the possibility of drug repurposing as SERCA inhibitors.

### 2.2. Pre-B Cell Acute Lymphoid Leukemia

Induction of SERCA3 expression could also be observed in a model of early B lymphocytic differentiation [139]. When various precursor-B acute lymphoblastic leukemia cell lines that express the E2A-PBX1 fusion oncoprotein are subjected to pharmacologically induced activation of protein-kinase C, SERCA3 expression is induced approximately five-fold [139]. This is accompanied by the induction of expression of several early B lymphoid differentiation markers such as CD19, CD20 or CD22, growth arrest, and the loss of expression of markers of immaturity (RAG-1, TdT, CD10) [139]. Induction of SERCA3 expression during the differentiation of leukemic precursor-B cells is a physiologically relevant phenomenon, because cell lines of mature B phenotype as well as normal non-activated mature B-lymphocytes express SERCA3 abundantly.

### 2.3. Megakaryocytic Differentiation

Induction of SERCA3 expression was also observed in leukemic megakaryoblastic cell lines treated with phorbol esters that induce megakaryocytic differentiation in these cells [189]. Induction of SERCA3 expression occurred in parallel with that of established megakaryocytic/platelet markers such as platelet glycoprotein IIIa [189]. Because SERCA3 is strongly expressed in normal mature megakaryocytes in situ, as well as in circulating platelets [124], this observation indicates that the induction of SERCA3 expression is part of the normal megakaryocytic differentiation program that is blocked in leukemic megakaryoblasts [190].

### 2.4. Down-Regulation of SERCA3 Expression In Vitro

Down-regulation of SERCA3 expression could be induced experimentally in models of cell activation and immortalization [143,147]. Activation of mature quiescent T lymphocytes by T cell receptor engagement leads to the induction of the expression of IL-2 receptor α chain and the simultaneous secretion of IL-2 by the cells. Secretion of IL-2 in parallel with the acquisition of functional, high affinity IL-2 receptors leads to auto/paracrine stimulation of T cells, and the cells acquire a blast-like, proliferative phenotype [58,191,192,193,194]. T cell activation can be modelled in vitro using Jurkat (JurE6-1) T lymphocytic cells treated simultaneously with a phorbol ester (PMA) and a calcium ionophore (ionomycin) [195]. Double treatment of JurE6-1 cells with PMA and ionomycin leads to an approximately 80% decrease of SERCA3 expression, while the expression of SERCA2 is slightly increased [147]. SERCA3 down-regulation, as well as IL-2 secretion, requires simultaneous treatment with PMA and ionomycin, and single treatments are ineffective. Importantly, SERCA3 down-regulation as well as the induction of IL-2 secretion in PMA plus ionomycin-treated cells are inhibited by the immunosuppressant cyclosporine-A [147] and the more specific calcineurin inhibitor FK-506 (tacrolimus; B.P., unpublished observation) [196].

SERCA3 down-regulation has also been reported in mature B lymphocytic cells following the establishment of latent infection with Epstein-Barr virus [143]. EBV infection of mature resting B-lymphocytes leads to the acquisition by the cells of an activated, immortalized, continuously proliferating phenotype [197,198]. Lymphoblastoid cell lines obtained by latent EBV infection of B cells resemble normal, antigen-activated proliferating lymphoblasts found in lymph node germinal centers, because several EBV-coded genes, such as EBNA-2 [199] and LMP1 [200], expressed in lymphoblastoid cell lines during EBV latency, constitutively activate various cellular signal transduction mechanisms also involved in the antigen-dependent activation of normal, uninfected B-lymphocytes [197,198,201]. Down-regulation of SERCA3 expression could be observed in Burkitt lymphoma cell lines chronically infected with an immortalization-competent, but not with an immortalization-deficient EBV strain [143]. SERCA3 expression was down-regulated also following the forced expression of LMP1 using an inducible expression vector in non-infected cells, whereas SERCA2 expression was not modified [143]. Resting mature normal B-lymphocytes express simultaneously SERCA2b and SERCA3-type calcium pumps. SERCA3 expression is, however, strongly decreased during normal B cell activation in situ, as seen using immunohistochemistry in lymph node follicle germinal centers [143]. These observations indicate that SERCA3 down-regulation is part of the immortalization program induced by EBV, a process that mimics the antigen-induced normal activation program of mature resting B lymphocytes.

### 2.5. SERCA3 Loss in Gastrointestinal Carcinomas

SERCA3 expression was investigated comparatively in normal colon epithelium and various benign and malignant colon tumors in situ using immunohistochemistry [126,142,202]. It was found that whereas normal colonic epithelium expresses high levels of SERCA3 protein, expression was variable in benign tumors (adenomas) and was decreased in adenocarcinomas [126,142,202]. The loss of SERCA3 expression was proportional to the histological grade of the malignant tumors, with a complete loss of expression in high grade, poorly differentiated adenocarcinomas in the colon [126,142]. Interestingly, unlike in neoplastic lesions, SERCA3 expression was maintained in hyperplastic colon polyps at levels comparable to normal epithelium [126]. Furthermore, whereas gastric surface epithelium expresses SERCA3 abundantly [126], in gastric cancer the loss of SERCA3 expression has been reported to be associated with shorter survival [203,204].

The APC/β -catenin/TCF4 molecular oncogenic pathway plays a central role in the transformation of colonic epithelial cells [205]. When this pathway was inhibited in colon carcinoma cells by the expression of a dominant negative version of the TFC4 protein [206], SERCA3 expression was induced, whereas the expression of the SERCA2b isoform was not modified significantly [142]. Moreover, the loss of SERCA3 expression in colon as well as gastric carcinoma cell lines could also be reversed in vitro by various cell differentiation-inducing treatments such as short chain fatty acids, other highly active histone deacetylase inhibitors or post-confluent growth, and this was accompanied by the induction of expression of various other phenotypic markers of epithelial differentiation [126,204,207]. Short-chain fatty acid-induced expression of SERCA3 was associated to decreased SERCA2b expression, increased resting cytosolic calcium levels and to a decreased thapsigargin-releasable intracellular calcium pool size in gastric carcinoma cells [126] in accordance with the lower calcium affinity of this enzyme. In addition, when the induction of colon cancer cell differentiation by post-confluent culture was conducted in the presence of various SERCA inhibitors, an enhanced differentiation response was observed [126], similarly to ATRA-induced differentiation of acute promyelocytic leukemia cells [74].

These data show that SERCA3 expression is part of the normal differentiation program of colonic epithelial cells, that SERCA3 expression in colon carcinoma is reversibly suppressed by the APC/β-catenin/TCF4 oncogenic pathway, that the inhibition of SERCA3 expression can be reversed by the induction of cell differentiation and that a functional crosstalk exists between SERCA function and the mechanisms that control differentiation in colon carcinoma cells.

### 2.6. Lung Cancer

SERCA3 expression in lung adenocarcinoma displayed similar characteristics to colon carcinoma. Whereas normal bronchial epithelium expresses high levels of SERCA3, expression in various lung adenocarcinoma tumors and cell lines was variable and often decreased or lost, and the induction of differentiation of the cell lines by short chain fatty acids or post-confluent growth led to enhanced SERCA3 expression, whereas the expression of SERCA2 was not modified significantly [140]. The induction of SERCA3 expression was accompanied by decreased calcium release from the SERCA-dependent intracellular pool, as studied with the pan-SERCA inhibitor thapsigargin in a calcium fluorimetry format using a genetically engineered calcium indicator [140], suggesting decreased ER calcium content, in accordance with the lower calcium affinity of the SERCA3 isoenzyme.

Decreased intra-ER calcium levels, as observed in differentiated gastric and lung carcinoma cells, are compatible with increased SERCA3 expression, for example, in a scenario in which SERCA3 overexpression is part of a process whereby a new, SERCA3-associated ER sub-compartment is created during cell differentiation, including the formation of new ER membrane surface area and ER lumen volume. At resting cytosolic calcium levels, calcium sequestration in this new part of the ER will be of lower intensity when compared to SERCA2b-associated regions, due to the weaker calcium affinity of SERCA3. Constitutive, passive calcium leak from the ER [208] will therefore not be re-sequestered in this new, SERCA3-associated ER sub-compartment at low cytosolic calcium levels, leading to a shift of the dynamic equilibrium of calcium release and reuptake towards increased net release from this region of a contiguous ER in a resting cell. Considering that the SERCA2b isoform will be nearly fully activated by calcium already at resting cytosolic calcium levels and thus unable to compensate for this calcium leakage, a new dynamic equilibrium will be established with higher cytosolic and lower intra-ER calcium concentrations, even when SERCA2b expression is maintained, rather than decreased in parallel with the induction of SERCA3 expression.

### 2.7. Breast Cancer

SERCA3 loss has also been observed in breast cancer tissue using immunohistochemistry [150]. Whereas normal lobular epithelial cells in breast acini express SERCA3 abundantly, SERCA3 expression is strongly decreased or lost in lobular carcinoma cells, as well as in non-malignant lobular lesions such as adenosis or lobular neoplasia in situ [150]. In invasive ductal breast carcinomas the loss of SERCA3 expression correlated with the Elston-Ellis grade of the tumors, as well as with individual components of this grading system such as marked nuclear pleomorphism and high proliferating index, and also with hormone receptor negative or triple negative (estrogen receptor-, progesterone receptor- and HER2-negative) status [150]. When taken together, these observations indicate that SERCA3 expression is already lost at the earliest histologically identifiable anomalies of acinar architecture and remains low during the further stages of lobular tumorigenesis, and that in ductal carcinomas the loss of SERCA3 expression is the most marked in highly proliferating, high nuclear grade, hormone receptor negative tumors. Conversely, the induction of SERCA3 expression by differentiation-inducing agents has been described on the mRNA level in various breast carcinoma cell lines [209,210], and the involvement of SERCA3 in progesterone-induced calcium signaling in breast cancer cell lines has also been reported [211].

### 2.8. Choroid Plexus

**Figure 3:**
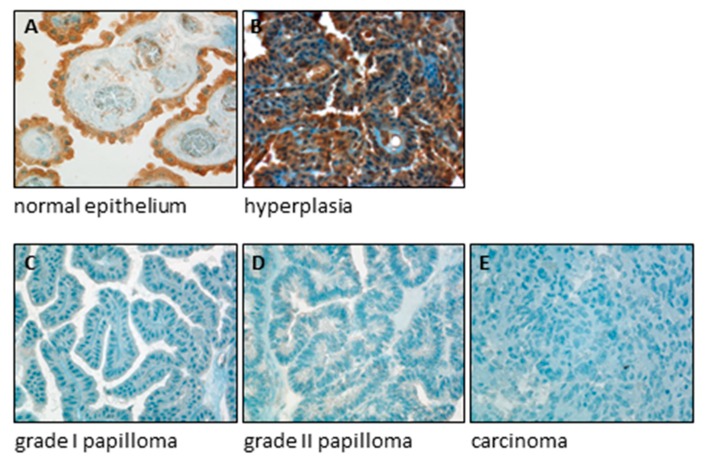
SERCA3 expression is lost in choroid plexus tumors. SERCA3 expression was investigated using immunohistochemistry with the 2H3 SERCA3-specific monoclonal antibody (Abnova) on deparaffinized, formalin-fixed sections of normal choroid plexus tissue (**A**), choroid plexus hyperplasia (**B**), grade I (**C**) and grade II papilloma (**D**) and choroid plexus carcinoma (**E**). Immunostaining was revealed using an avidin-biotin-peroxydase immunolabeling system with 3,3′diaminobenzidine as chromogen. Slides were counterstained with hematoxylin (blue). Whereas normal choroid plexus epithelial cells (**A**) and hyperplastic epithelium (**B**) express SERCA3 abundantly (brown coloration), expression in grade I and grade II papillomas (**C**,**D**) as well as in carcinoma (**E**) is barely detectable or absent. Original magnification: ×40.

Located in cerebral ventricles, the choroid plexus is involved in the production and the homeostatic regulation of the composition of cerebrospinal fluid [212]. The choroid plexus epithelium accomplishes specialized trans-cellular transport functions of water, ions, vitamins, other nutrients and metabolites between the blood and the cerebrospinal fluid [212]. Neoplastic lesions of the choroid plexus epithelium can be divided according to their malignant potential. Whereas grade I papillomas are fully benign, grade II papillomas are of low malignant potential, whereas choroid plexus carcinomas are fully malignant [213,214,215,216]. It has been shown that whereas normal choroid plexus epithelium expresses high amounts of SERCA3 protein as detected using immunohistochemistry, expression is strongly decreased in grade I and II papillomas and undetectable in carcinomas [146]. As shown in Figure 3, in contrast with tumors, SERCA3 expression in choroid plexus hyperplasia, which is a non-neoplastic lesion [217,218], was high, similarly to fully differentiated normal choroid plexus epithelium [146]. In addition, treatment of primary normal choroid plexus epithelial cells with short-chain fatty acid-type differentiation-inducing agents in vitro led to the induction of the expression of SERCA3 [146]. These data show that SERCA3 expression is part of the normal gene expression program of mature choroid plexus epithelial cells, that SERCA3 expression is lost already in benign choroid plexus tumors and that SERCA3 immunohistochemistry may be useful to distinguish hyperplastic lesions from papillomas in the choroid plexus.

## 3. Conclusions

Observations made on multiple different tissue and cell types and their tumors of various degrees of malignancy show that whereas several normal epithelial cell types, as well as cells of hematopoietic origin express simultaneously SERCA2b and SERCA3-type calcium pumps, SERCA3 expression is decreased or lost in corresponding tumors or leukemias. The fact that SERCA3 loss has been found in a wide range of tumors of different histological origin indicates that deficient SERCA3 expression is a widespread phenomenon in neoplasia. The degree of SERCA3 down-regulation correlated with the degree of malignancy of various tumors and was, on the other hand, maintained in hyperplastic lesions. These observations show that SERCA3 may serve as a useful new marker for the pathologist for the immunohistochemical analysis of tumors and that the SERCA3 status of tumors may convey clinically relevant information.

Differentiation of tumor cells induced in vitro by the targeted inhibition of various molecular oncogenic mechanisms, or using differentiation-inducing agents such as short chain fatty acids and their analogues, leads to enhanced SERCA3 expression, in parallel with the induction of the expression of other phenotypic markers of differentiation. SERCA3 expression is part of the normal differentiation process of several cell types, whereby the cells acquire an ER homeostatic and signaling configuration required for the specialized effector functions of the fully differentiated, specialized cell, a process blocked in tumors. Induction of SERCA3 expression during cell differentiation is associated to changes of ER calcium homeostasis. It may be hypothesized that during cell differentiation, the extension by a newly formed, SERCA3-associated ER sub-compartment of an originally SERCA2b-expressing ER may increase net passive background calcium leakage [208] from the organelle at resting cytosolic calcium concentrations, due to the lower calcium affinity of SERCA3. This may lead to decreased intra-ER calcium levels and more cytosolic calcium, potentially sensitizing the cell to calcium-dependent cytosolic processes at a resting state, as well as to capacitative calcium influx and calcium-activated effector functions upon stimulation by extracellular ligands.

The direct pharmacological inhibition of SERCA-dependent calcium sequestration can enhance cell differentiation and modulate the expression of oncoproteins, depending on cell type. This indicates that a functional crosstalk exists between SERCA-dependent calcium sequestration in the ER and various molecular mechanisms that control cell differentiation. Absence of SERCA3 in tumors points to a previously unknown link between cellular calcium homeostasis, tumorigenesis and the loss of terminal differentiation of malignant cells.

The highly inducible nature of SERCA3 expression indicates that SERCA-dependent calcium sequestration is not static. Changes of the absolute amount of SERCA enzymes as well as changes of SERCA2/SERCA3 expression ratios can occur readily in a cell. The modulation of SERCA levels constitutes a unique mechanism whereby the cell can adjust and modulate its calcium homeostasis depending on its phenotype.

Interestingly, changes of SERCA isoenzyme expression levels are not the sole modifications of cellular calcium transport that occur during the differentiation of malignant cells. The expression of PMCA-type calcium pumps also undergoes significant isoform-specific changes during the differentiation of gastric and colon carcinoma cells [219,220,221], PMCA isoenzyme composition of various molecular and histological types of breast carcinoma displays tumor type-specific characteristics [222,223,224,225,226], the PMCA isoenzyme composition of estrogen receptor-positive breast cancer cells is modulated by estradiol [227], and normal breast epithelium also undergoes important changes in terms of PMCA isoenzyme expression during lactation [194,228,229,230,231,232,233]. Another interesting observation is that in BRAF mutant melanoma cells a specific PMCA isoform (PMCA4b) is down-regulated in a BRAF/ERK-dependent manner, and the reintroduction of this isoform can reduce the migratory and metastatic activities of these cells [234,235].

These observations taken together indicate that the cellular calcium homeostatic network can undergo complex modifications during oncogenesis or cell differentiation, which may lead to profound changes in the way a cell generates and processes calcium signals and how it ultimately responds to them. The various in vitro differentiation models discussed in this work may constitute a useful platform for further, integrated functional studies aimed at understanding the relevant differences that distinguish normal cells from neoplastic ones in terms of their calcium homeostasis and signaling, as well as studying the process of functional specialization and differentiation of the ER.

The selective association of SERCA2b and SERCA3 with functionally distinct intracellular calcium pools has been described [180,236,237,238,239], and SERCA2b-and SERCA3-associated calcium pools can be localized at separate regions in a polarized epithelial cell [239]. Considering the differences in their calcium affinities discussed earlier, the differential distribution of SERCA2b and SERCA3 can lead to situations where calcium uptake into the ER happens in a non-uniform manner. In a resting cell, calcium will be taken up into the ER mainly by SERCA2b, the higher calcium affinity isoenzyme, whereas during a calcium activation event, SERCA3-dependent calcium pools will also be replenished. This can lead to complex cytosolic calcium signals and calcium oscillations [37,56,176,240], the frequency and amplitude of which can encode information allowing the selective activation of distinct downstream calcium-activated enzymes [30,59,241,242]. In addition, it is tempting to speculate that the non-homogeneous distribution of SERCA2b and SERCA3 within a contiguous ER membrane network can lead to intra-luminal calcium ion gradients and calcium ion migration, which may serve as an organizing force for the distribution, state of aggregation and regulation of activity of, for example, intra-ER calcium-binding chaperone proteins [243]. Changes of SERCA composition of a cell can thus have far-reaching consequences for the structure and the function of the ER.

The regulation of SERCA expression depending on cell type and the state of cellular differentiation, activation or transformation constitutes a previously unrecognized component of cellular calcium homeostasis and signaling. Shifts in SERCA isoenzyme levels need to be taken into account when an integrated investigation of cellular calcium homeostasis is attempted, in particular when cells of various phenotypes (for example, normal vs. neoplastic, undifferentiated vs. differentiated or quiescent vs. proliferating) are investigated in a comparative manner. This will contribute to the identification of new levels of complexities of intracellular calcium homeostasis and signaling and their connections to oncogenesis.

## Abbreviations

APL	acute promyelocytic leukemia
ATRA	all-*trans* retinoic acid
DAG	diacylglycerol
E2A	E2A immunoglobulin enhancer-binding factor E12/E47
EBNA-2	Epstein-Barr virus nuclear antigen 2
EBV	Epstein-Barr virus
ER	endoplasmic reticulum
ERK	extracellular signal-regulated kinase
IL-2	interleukin-2
IP3	inositol 1,4,5-trisphosphate
LMP1	Epstein-Barr virus latent membrane protein 1
MCU	mitochondrial calcium uniporter
NCX	sodium/calcium exchanger
PBX1	pre-B-cell leukemia transcription factor 1
PIP2	phosphatidylinositol 4,5-bisphosphate
PLC	phospholipase C
PMA	phorbol 12-myristate 13-acetate
PMCA	plasma membrane calcium ATPase
PML	promyelocytic leukemia protein
RAG-1	recombination activating gene 1
SERCA	sarco/endoplasmic reticulum calcium ATPase
SPCA	secretory pathway calcium ATPase
STIM	stromal interaction molecule
TdT	terminal deoxynucleotidyl transferase
